# Process and effects of a community intervention on malaria in rural Burkina Faso: randomized controlled trial

**DOI:** 10.1186/1475-2875-7-50

**Published:** 2008-03-25

**Authors:** Bocar Kouyaté, Florent Somé, Albrecht Jahn, Boubacar Coulibaly, Jaran Eriksen, Rainer Sauerborn, Lars Gustafsson, Göran Tomson, Heiko Becher, Olaf Mueller

**Affiliations:** 1 Centre de Recherche en Santé de Nouna, Nouna, Burkina Faso, Africa; 2 Department of Tropical Hygiene and Public Health, University of Heidelberg, Germany; 3 Karolinska Institute, Stockholm, Sweden

## Abstract

**Background:**

In the rural areas of sub-Saharan Africa, the majority of young children affected by malaria have no access to formal health services. Home treatment through mothers of febrile children supported by mother groups and local health workers has the potential to reduce malaria morbidity and mortality.

**Methods:**

A cluster-randomized controlled effectiveness trial was implemented from 2002–2004 in a malaria endemic area of rural Burkina Faso. Six and seven villages were randomly assigned to the intervention and control arms respectively. Febrile children from intervention villages were treated with chloroquine (CQ) by their mothers, supported by local women group leaders. CQ was regularly supplied through a revolving fund from local health centres. The trial was evaluated through two cross-sectional surveys at baseline and after two years of intervention. The primary endpoint of the study was the proportion of moderate to severe anaemia in children aged 6–59 months. For assessment of the development of drug efficacy over time, an *in vivo *CQ efficacy study was nested into the trial. The study is registered under  (ISRCTN 34104704).

**Results:**

The intervention was shown to be feasible under program conditions and a total of 1.076 children and 999 children were evaluated at baseline and follow-up time points respectively. Self-reported CQ treatment of fever episodes at home as well as referrals to health centres increased over the study period. At follow-up, CQ was detected in the blood of high proportions of intervention and control children. Compared to baseline findings, the prevalence of anaemia (29% vs 16%, p < 0.0001) and malaria parameters such as prevalence of *P. falciparum *parasitaemia, fever and palpable spleens was lower at follow-up but there were no differences between the intervention and control group. CQ efficacy decreased over the study period but this was not associated with the intervention.

**Discussion:**

The decreasing prevalence of malaria morbidity including anaemia over the study period can be explained by an overall increase of malaria prevention and treatment activities in the study area. The lack of effectiveness of the intervention was likely caused by contamination, pre-existing differences in the coverage of malaria treatment in both study groups and an unexpectedly rapid increase of resistance against CQ, the first-line treatment drug at the time of the study.

## Background

At least one million annual malaria deaths occur among young children in rural sub-Saharan Africa (SSA). Most of these deaths are in populations with little access to health services [1-4] In such areas, home treatment with chloroquine (CQ), antipyretics and traditional remedies is the most frequent response of caretakers to fever episodes in children [4-6]. However, due to the increasing resistance against CQ in most countries in SSA together with limited access to modern health services, poor quality of such services, low compliance with treatment schemes and poor quality of drugs sold at markets, the community effectiveness of malaria treatment is very low [6-13].

As malaria treatment provided through formal health services is currently not a sufficiently effective strategy for malaria control in rural SSA, interventions aiming at improving malaria home treatment by main caretakers, usually the mothers, may be considered as a complementary strategy. There is some evidence that improved home management of malaria in young children of SSA will result in earlier and more effective treatment with consequently reduced morbidity and mortality [14,15].

This project is an EU INCO-DEV funded collaboration between the Heidelberg University (Germany), Karolinska Institute (Sweden), Muhimbili University College of Health Sciences (Tanzania) and Centre de Recherche en Santé de Nouna (Burkina Faso) called MAMOP project (Improving the management of childhood **MA**laria: an experiment to bridge the gap between **MO**thers and health care **P**roviders). It is a controlled malaria community intervention with a pre-post design conducted in rural Burkina Faso and Tanzania in 2002 – 2004. The overall objective of the MAMOP study was to evaluate the feasibility and effectiveness of an intervention aimed at improving case management of malaria in underfive children through primary caretakers in collaboration with local women groups and existing health centres.

## Methods

### Study area

**Figure 1:**
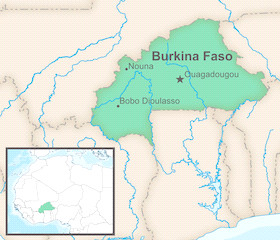
Map of Burkina Faso.

The study was implemented in the rural part of the research zone of the Centre de Recherche en Santé de Nouna (CRSN) in Nouna Health District, north-western Burkina Faso (Figure 1). The Nouna area is a dry orchard savannah, populated mainly by subsistence farmers of different ethnic groups. Malaria is holoendemic but highly seasonal, and the transmission intensity varies between 100 and 1000 infective bites per person and year between study villages [16,17]. Formal health services in the study area are provided by a limited number of rural health centres and the district hospital in Nouna town [18]. Village-based health centres are usually equipped with two nurses and one mid-wife and do outreach work in the surrounding 7–10 villages under their responsibility. Malaria control is mainly based on home treatment with CQ, which has been shown to be still sufficiently effective in 2001, and on malaria prophylaxis for pregnant women [4,19,20]. Untreated mosquito nets have been used in the area for a long time, but insecticide-treated nets (ITN) were only recently introduced in the frame of an effectiveness study [21,22]. Communities in the study area have been shown to be quite well organized with regard to risk sharing mechanisms. In particular women groups with a focus on mutual agricultural support traditionally exist in all villages [23].

### Study design

**Figure 2:**
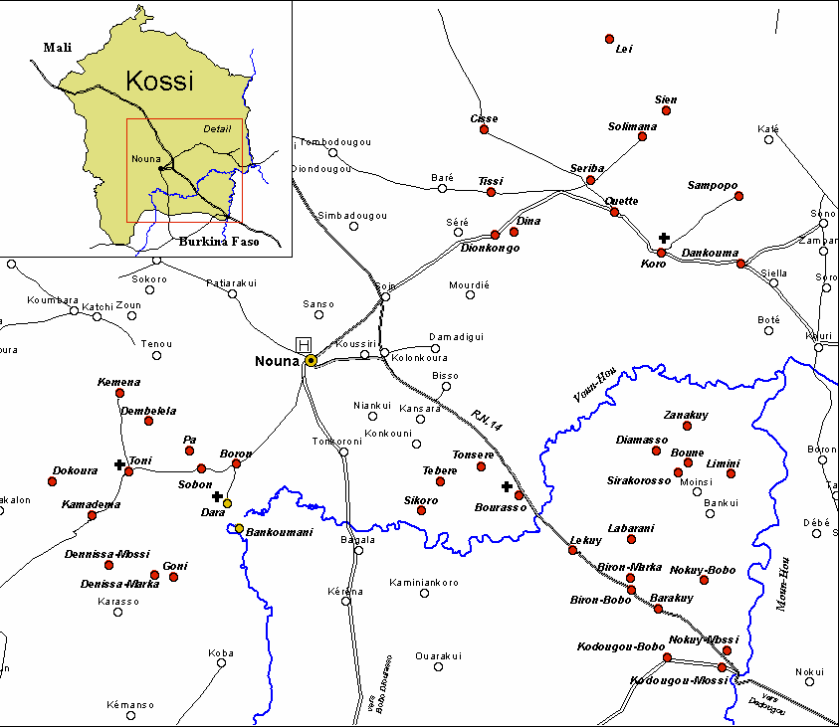
Map of the Nouna study area.

The study was designed as a cluster-randomized controlled effectiveness trial. Using the data base of the Nouna Demographic Surveillance System (DSS) [24], villages were selected by lottery (OM, AJ) until an approximate sample size of 1.200 households per study arm was achieved. As a result, six (Pa, Toni, Kemena, Denissa-Mossi, Boune, Lekuy) and seven (Bankoumani, Kamadena, Labarani, Barakuy, Dankoumana, Soulemana, Tissi) villages were randomly assigned to the intervention and control arms respectively (Figure 2).

The primary outcome of the study was the proportion of moderate to severe anaemia (haematocrit ≤ 24%) in children aged 6 to 59 months. Secondary outcomes were prevalences of fever, malaria, of palpable spleens (Hackett score ≥ 2), of other illnesses, mean species-specific number of blood films positive for malaria parasites, mean species-specific malaria parasite densities, mean haematocrit values and mean weight. Finally, *in vivo *CQ efficacy was measured in all study children with uncomplicated falciparum malaria at baseline and at follow-up using a modified version of the standard WHO protocol [25].

To be able to detect a 10% difference in anaemia prevalence between the intervention and control group with 80% power and at a significance level of 5%, and assuming a prevalence in the control group of 20%, and a conservative design factor of 2.5, 992 under-five children were required [26]. As the households were used as units of analysis and as around 80% of all households include an under-five child, about 1200 households had to be sampled.

### The intervention

The intervention was targeted at three groups: health workers (nurses) from five peripheral health centres (Toni, Dara, Bourasso, Lekuy, Koro), women group leaders, and the main care takers (usually the mothers) of preschool children. The community members of the six intervention villages selected a total of 70 women group leaders, two by sub-village (sub-village numbers ranged from 3–9 per village). Inclusion criteria for group leaders used by the communities were permanent residency in the sub-village, age 30–50 years, honesty, and respect by the community. The main components of the intervention were:

• Training of health staff, women group leaders and mothers

• Sensitisation of the communities

• Drug supply to women group leaders, revolving fund

• Supervision of health workers and women group leaders

• Intervention information system.

**Figure 3:**
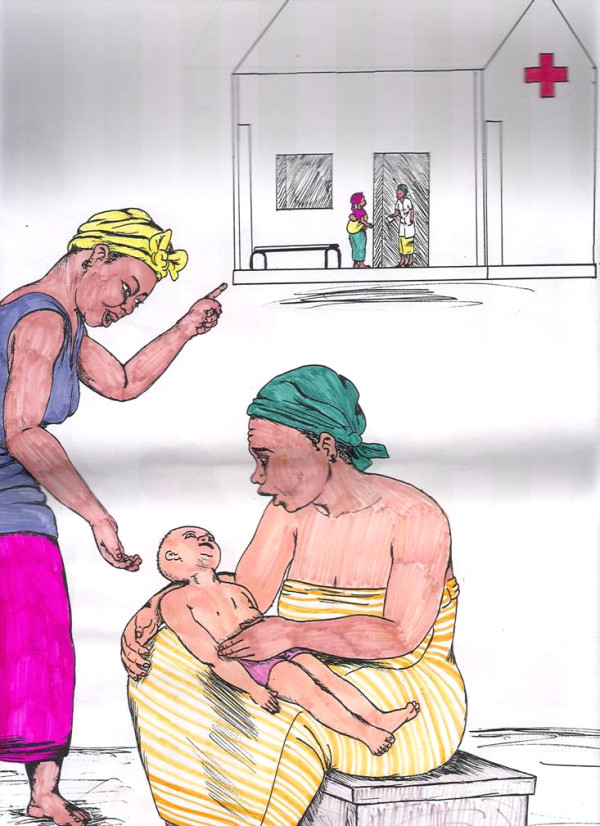
Pictoral chart example: referral of severely sick children to the health centre.

**Figure 4:**
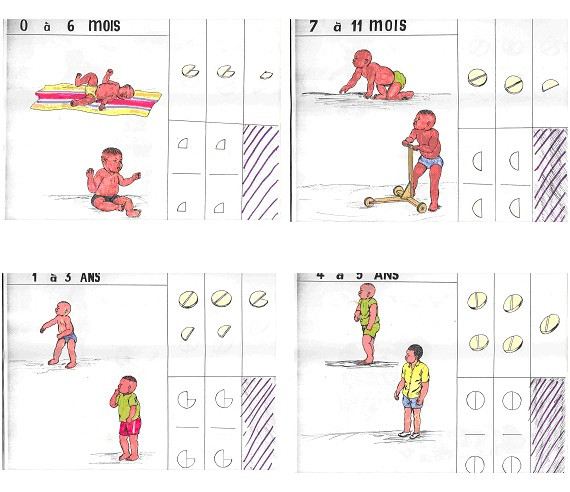
Pictoral chloroquine treatment guidelines by age group.

**Figure 5:**
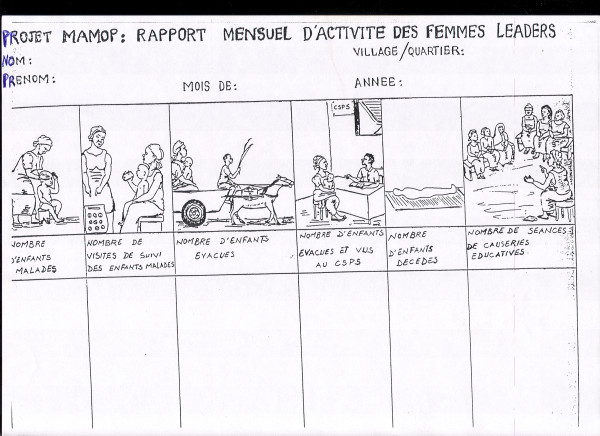
Pictoral chart example: intervention information system.

A five days training course for the health workers of participating health centres was conducted by one of the investigators (FS) together with the District Medical Officer and included an update on malaria case management and an introduction to adult non formal education methods. Afterwards, all women group leaders were trained in respective peripheral health centres by the health workers under the supervision of the investigators for two days on all relevant aspects of malaria knowledge and management including referral criteria. The training included discussions as well as practical sessions using locally produced pictorial sensitisation material and role play, with one refresher course over the study period (Figures 3, 4, 5). After this was completed, a sensitisation campaign was done in all intervention villages. Thereafter, the women group leaders under the supervision of their local health workers trained an average of 15 mothers for half a day in their sub-villages on correct malaria management.

All women group leaders were regularly provided with CQ and paracetamol from the essential drug stock of Nouna Health District. Pre-packed tablets have been shown to be better for ensuring compliance compared to loose tablets in African countries including Burkina Faso [14,27,28]. Thus, CQ and paracetamol were pre-packed in plastic bags in four age-specific doses (0 – 6 months, 7 – 11 months, 1 – 3 years, 4 – 5 years) each with a specific colour and containing pictorial guidelines according to national malaria treatment guidelines (Figures 3, 4, Table 1).

**Table 1:** Drug package (chloroquine + paracetamol) prices by provider and age group

Age groups	Prices to women leaders	Prices to caretakers
0 – 6 months	15 FCFA (0.03 US $)	30 FCFA (0.06 US $)
7 – 11 months	20 FCFA (0.04 US $)	40 FCFA (0.08 US $)
1 – 3 years	30 FCFA (0.06 US $)	60 FCFA (0.12 US $)
4 – 5 years	40 FCFA (0.08 US $)	80 FCFA (0.16 US $)

Mothers with febrile children were advised to go to the house of women group leaders for early treatment. Children were treated with a total dose of 25 mg/kg CQ over a period of three days (first and second day: 10 mg/kg, third day: 5 mg/kg). In addition, all children received standard doses of paracetamol (10 mg/kg every 12 hours) over the first two days. Women group leaders were advised to directly supervise the first dose of CQ and paracetamol, and to visit the sick child again on the second and third day. In case of danger signs at any time point or ongoing fever at the end of the treatment time, the women group leaders had to refer the child to the health centre.

At the beginning of the intervention, a six months stock of drugs was provided free of charge to the women group leaders, who had to afterwards renew their stock by buying new drugs from the health workers. Drugs were sold by the women group leaders to the mothers/caretakers at prices which allowed them to make a small profit as an incentive (table 1). The health workers visited the sub villages monthly for supervision with a standardized checklist, collection of forms and for drug provision. Overall supervision and if necessary – specific support – was carried out by the investigators every month for the first three months and thereafter every three months.

The intervention was monitored through a specific intervention information system which used forms based on pictorial charts, which were filled in every month by the women group leaders (figure 5). The first form collected information on (1) the number of malaria cases managed, (2) the number of home visits done, (3) the number of cases referred to the health facilities, (4) the number of children who had visited the health facilities, and (5) the number of children who had died. The second form reported the time between illness onset and initiation of the treatment. The third form reported the number of pre-packaged drug bags used for each age group every month.

Information about the process of the intervention in health facilities was gathered through specific data collection tools (supervision report, pre-packaged drugs inventory list) as well as through the routine health information system (health centres registers, patients clinical files, referrals and counter referrals sheets).

The intervention had been pre-tested in three villages outside the study area. For the main study, training of health staff and women group leaders took place from February until June 2003. The intervention itself was fully implemented from July 2003 until October 2004, thus covering two rainy seasons.

### Study evaluation

A baseline survey took place in September/October 2002, and a follow-up survey in September/October 2004.

All households with children below 5 years of age were included into the two surveys. In households with more than one eligible child, the survey child was chosen by lottery. All survey children were examined by the study physician (FS). Children found ill during the surveys were treated according to national guidelines. Those fulfilling the criteria for uncomplicated falciparum malaria (fever + ≥ 5.000 *Plasmodium falciparum *parasites per μl) were followed up for *in vivo *drug efficacy testing over a two weeks period. The WHO definitions for early treatment failure (ETF), late clinical failure (LCF), late parasitological failure (LPF) and adequate clinical and parasitological response (ACPR) were applied [25].

All children included into the *in vivo *drug efficacy study received standard doses of CQ and paracetamol over three days. Administration of study medications was directly supervised by the study team on day 0 and by village-based field workers on day 1 and day 2. In case of vomiting within one hour after the study medication, the medication was repeated. No drugs were allowed as concomitant treatment. Over the 14 days follow-up period, study children were seen daily by the field workers, who checked them for danger signs, measured their axillary temperature with a thermometer, and took finger prick blood samples. In case of clinical or parasitological failure, children were given single-dose pyrimethamine-sulfadoxine rescue treatment (taken from the essential drug store of Nouna Health District) according to national guidelines. Children with danger signs were referred to the health centre.

Treatment procedures were followed through documentation of self-reported behaviour during surveys as well as through CQ determination in blood samples.

### Laboratory investigations

From all children seen at the baseline and follow-up survey, a finger-prick blood sample was taken. From this, thin and thick blood smears were prepared for malaria diagnosis while anaemia determination was done through measurement of haematocrit values with a portable microhaematocrit centrifuge (Compur Microspin, Bayer Diagnostics, Germany).

For those children included into the CQ efficacy study, additional finger-prick blood samples were taken on day 2, day 3, day 7, day 14, and on any other day the child presented with symptoms during follow up according to standard procedures [25]. Moreover, pre-treatment filter paper blood samples were taken from every child and randomly distributed in equal proportions for determination of chloroquine content and chloroquine resistance markers. Concentration of chloroquine and its metabolite desethylchloroquine were measured using high pressure liquid chromatography at Karolinska Institute in Stockholm.

Blood films were kept in closed slide boxes until they were transported to the CRSN laboratory in Nouna town. After Giemsa-staining, all films were examined by experienced laboratory technicians. Thick and thin blood films were analysed for the species-specific parasite density per μl by counting against 200 white blood cells and multiplying by 50. Slides were declared negative if no parasites were seen in 400 fields on the thick film. For quality control a 10% random sample of blood films is regularly cross checked at the laboratory of the Heidelberg School of Tropical Medicine [21].

### Statistical analysis

To assess the effect of the intervention, logistic regression was used. In separate models, anaemia (haematocrit ≤ 24%), fever (≥ 37.5°C), spleen enlargement (Hackett score ≥ 2), clinical malaria (fever + ≥ 5000 parasites/μl), and malaria parasitaemia (>0 parasites/μl) were considered as binary outcomes. Age (continuous), weight (continuous) and ethnic group (4 groups: Bobo, Dafing, Mossi, other) were considered as co-variables for adjustment in the model. The distribution of the outcome variables in intervention and control group was compared at baseline to check adequacy of randomization. Fisher exact test was used to compare rates. The analysis was performed with SAS release 8.02 (SAS^® ^Institute Inc, Cary, NC, USA).

### Ethical aspects

The protocol was approved by the local Ethics Committee in Burkina Faso. Community consent was sought during village meetings in all study villages. During the following visits to individual households, caretakers were asked for their oral consent after having received detailed information from the study physician about all risks and benefits of the study. They were clearly informed that they could withdraw from the study at any time and without disadvantage. All children in the specified age group found to be ill during cross-sectional surveys or during follow-up in case of the CQ resistance study received free treatment in the village or were referred to Nouna hospital if indicated.

## Results

### Study group characteristics

**Figure 6:**
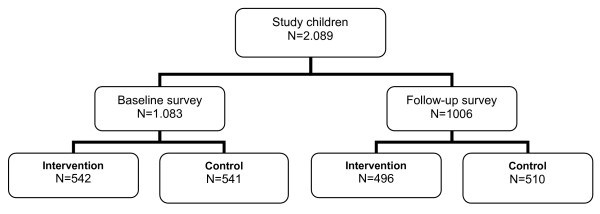
MAMOP study flow chart.

A total of 1,083 children (542 from intervention and 541 from control villages) and a total of 1006 children (496 from intervention and 510 from control villages) were included into the study at baseline and follow-up time points respectively (Figure 6).

Table 2 shows demographic characteristics of children from both study groups at baseline and follow-up time points. Comparing children from intervention and control clusters at baseline and follow-up, there were no significant differences with regard to age and sex. However, intervention and control clusters differed much with regard to the presence of the Bobo and Marka ethnicity. Comparing children from the intervention and control clusters over time, the samples did not differ significantly with the exception of follow-up children having been slightly older in both groups at follow-up.

**Table 2:** Demographic, clinical and parasitological characteristics of study children at baseline and follow-up time points.

	Baseline survey	Baseline survey	Follow-up survey	Follow-up survey
	Intervention (n = 542)	Control (n = 541)	Intervention (n = 496)	Control (n = 510)

Ethnicity (%)				
Marka	99 (18)	312 (58)	100 (20)	305 (60)
Bobo	301 (56)	116 (21)	280 (56)	96 (19)
Peulh	33 (6)	35 (6)	17 (3)	35 (7)
Mossi	81 (15)	51 (9)	79 (16)	44 (9)
Samo	18 (3)	21 (4)	13 (3)	26 (5)
Other	10 (2)	6 (1)	7 (1)	4 (1)
Median age (months)	31	30	35	35
Median age (range)	(5–56)	(4–56)	(5–60)	(5–60)
Male/female	285/257	266/275	238/258	267/243

**Figure 7:**
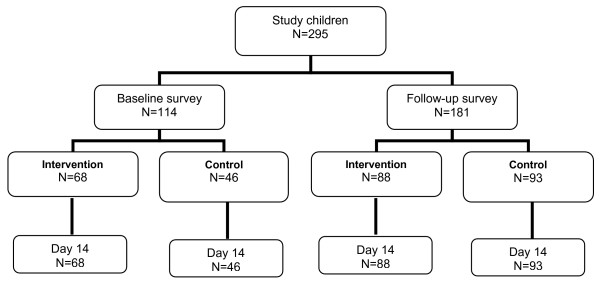
Chloroquine efficacy study flow chart.

A total of 114 children (68 from intervention and 46 from control villages) and a total of 181 children (88 from intervention and 93 from control villages) were included into the CQ efficacy study at baseline and follow-up time points respectively (Figure 7). The difference in number of children from this sub-study at baseline and follow-up is explained by the fact that for logistical reasons, the recruitment of children at baseline was only in four villages (two intervention, two control), while at follow-up, it was in nine villages (five intervention, four control). All four villages sampled at baseline were again sampled at follow-up. There were no major differences in baseline demographic, clinical or parasitological characteristics between survey children (Table 3). All study children completed the 14 days follow-up.

**Table 3:** Demographic, clinical and parasitological characteristics of children included into the chloroquine efficacy study at baseline and follow-up time points

	Baseline survey	Baseline survey	Follow-up survey	Follow-up survey
	Intervention (n = 68)	Control (n = 46)	Intervention (n = 88)	Control (n = 93)

Mean age, range (months)	30.1; 6–58	33.5; 12–60	25.1; 6–58	27.7; 6–58
Male	35	21	46	45
Mean weight, range (kg)	11.1; 6.5–16.9	11.2; 5.9–17.3	10.1; 5.9–18.3	10.3; 5–16
Mean temperature, range (°C)	38.0; 37.5–39.5	38.0; 37.5–39.4	38.1; 37.5–40.1	38.2; 37.5–40.5
Mean number *P. falciparum *trophozoites, range	19.826, 5.000–67.000	24.541; 5.000–158.000	33.448; 5.000–174.500	21.063; 5.000–101.000

### Compliance with the intervention

Some 12.500 pre-packaged malaria treatments were sold in the intervention villages over the period July 2003 until October 2004. Table 4 shows the data on self-reported compliance in study children during baseline and follow-up surveys. The number of self-reported fever episodes (fever during last two days) was lower during the baseline survey compared to the follow-up survey (29% vs 52%), without differences between study groups. At baseline, more children in the control villages compared to intervention villages reported having treated these fever episodes with CQ at home (64% vs 35%), but this was reversed at follow-up (60% vs 72%). At follow-up, women group leaders were reportedly consulted for the management of CQ home treatment episodes in 88% in the intervention group but also in 9% of the control group. The proportion of fever episodes treated at formal health services was very low at baseline and increased over the study period (2% vs 11%). There were no differences in this treatment pattern at baseline (2% vs 2%), but the increase was higher in the intervention compared to the control villages during follow-up (15% vs 8%).

**Table 4:** Self-reported treatment procedure information in study children during baseline and follow-up surveys

	Baseline survey	Baseline survey	Follow-up survey	Follow-up survey
	Intervention (n = 542)	Control (n = 541)	Intervention (n = 496)	Control (n = 510)

Fever last 2 days (%)	179/542 (33)	132/541 (24)	241/496 (49)	282/510 (55)
Treatment of fever episode				
- with CQ at home (%)	62/179 (35)	85/132 (64)	172/241 (72)	168/282 (60)
- at health centre (%)	4/179 (2)	2/132 (2)	36/241 (15)	23/282 (8)
Involvement of women group leaders in CQ treatment	-	-	151/172 (88)	15/168 (9)

Due to technical problems, no measurements of CQ blood levels were possible during the baseline survey. During the follow-up survey, CQ was detected in the blood of 33/43 (77%) and 37/43 (86%) while the median CQ blood level (range) was 212 nmol/L (23–26.878) and 407 (23–13.734) nmol/L in intervention and control group children respectively.

### Effects of the intervention on primary and secondary outcomes

Table 5 gives the primary and secondary study outcomes except the CQ efficacy results. The prevalence of anaemia, fever, splenomegaly and *P. falciparum *parasitaemia but not of falciparum malaria was significantly lower during the follow-up compared to the baseline survey, but there were no significant differences between intervention and control group. A multivariate analysis showed no effect of the intervention on any of the outcome variables considered. However, there was a highly significant improvement regarding the prevalence of anaemia, fever, malaria, *P. falciparum *parasitaemia and splenomegly at the follow-up survey independent of the intervention (p < 0.001).

**Table 5:** Effects of the intervention on study outcomes

	Baseline survey	Baseline survey	Follow-up survey	Follow-up survey	p^§^
	Intervention (n = 542)	Control (n = 541)	Intervention (n = 496)	Control (n = 510)	

Anaemia* (%)	152 (28)	162 (30)	83 (17)	74 (15)	0.32
Fever prevalence** (%)	201 (37)	189 (35)	143 (29)	142 (28)	0.40
Falciparum malaria prevalence*** (%)	87 (16)	85 (16)	64 (13)	71 (14)	0.45
Spleen enlargement**** (%)	108 (20)	79 (15)	22 (4)	19 (4)	0.08
*P. falciparum *parasitaemia (%)	455 (84)	438 (81)	379 (76)	366 (72)	0.05
Range	80–100.000	40–129.000	50–180.000	100–108.000	
Median *P. falciparum*	5.000	5.000	3.000	3.000	
Median haematocrit (range)	28 (12–40)	28 (14–40)	30 (16–42)	30 (10–40)	
Median weight (kg)	11.2	11.1	11.1	11.6	

* haematocrit ≤ 24%, ** ≥ 37.5°C, *** ≥ 37.5°C + ≥ 5.000 parasites/μl, ****Hackett ≥ 2, ^§ ^p-value for effect of intervention on study outcomes adjusted for time, age, weight and ethnic group

The results of the *in vivo *CQ efficacy study are given in table 6. At baseline, the overall day 14 ACPR was 76/114 (67%), without differences between children from intervention and control villages. At follow-up, an ACPR occurred in 46/88 (52%) of intervention group children and in 41/93 (44%) of control group children. After controlling for baseline variables, there was a significant effect of time (OR 0.4, 95% CI 0.2–0.7), but not of the intervention (OR 1.4, 95% CI 0.8–2.5) on ACPR.

**Table 6:** Chloroquine efficacy in young children of rural Burkina Faso in intervention and control communities

	Baseline survey	Baseline survey	Follow-up survey	Follow-up survey	p-value*
	Intervention (n = 68)	Control (n = 46)	Intervention (n = 88)	Control (n = 93)	

ETF (%)	8 (12)	3 (7)	13 (15)	12 (13)	0.88
LCF (%)	4 (6)	5 (11)	4 (5)	13 (14)	0.04
LPF (%)	11 (16)	7 (15)	25 (28)	27 (29)	0.99
ACPR (%)	45 (66)	31 (67)	46 (52)	41 (44)	0.30

ETF = Early Treatment Failure; LCF = Late Clinical Failure; LPF = Late Parasitological Failure; ACPR = Adequate Clinical and Parasitological response.

*Comparison of proportions at follow-up survey (Fisher exact test)

## Discussion

This study was designed as a cluster-randomized controlled trial with a sufficient sample size to show intervention effects of public health importance on malaria parameters. The intervention was shown to be feasible under programme conditions and the uptake was documented by an increase of malaria treatment with CQ in the intervention households and by an increase of referrals to the local health centres. Unfortunately, at baseline the use of CQ was higher in the control compared to the intervention villages which points to the fact that intervention and control area differed with regard to treatment behaviour. However, although CQ treatment increased in the intervention villages it was observed to a similar degree in the control villages. This is to a large degree explained by the high degree of pre-existing CQ treatment in control villages. Moreover, contamination could have occurred by communication between the villagers of both study arms but also by local health workers who partly were responsible for both intervention and control villages. Such an interpretation is also supported by the fact that some 10% of women in control villages also received support from women group leaders. A further explanation for the high degree of CQ treatment in the control villages is a possible contamination by a parallel community-based CQ-distribution project in the area. In this GTZ (Gesellschaft für Technische Zusammenarbeit) – supported reproductive health project, women of 18 villages neighbouring the MAMOP study area received contraceptives but also CQ through trained volunteers. This unforeseen additional contamination has likely contributed to making the control zone not a real control zone.

The main findings from the study were significant reductions in the prevalence of anaemia, *P. falciparum *parasitaemia and spleen enlargement over time, but without differences between study groups. The differences in the prevalence of anaemia and malaria parameters between the baseline survey in 2002 and the follow-up survey in 2004 are likely caused by a higher use of CQ in the study area. This hypothesis is supported by the finding of high CQ blood levels both in intervention and control children during follow-up. However, the effects of another community based malaria study which was implemented in all villages of the rural CRSN study and during which all newborn children received insecticide-treated mosquito nets (ITN) free of charge may also play a role in this overall dynamic [21]. As the enrolment of such study children took place from mid-2000 until the end of 2002, increasing protection of under five children with ITN is a likely additional explanation for the observed reductions of malaria morbidity in both the intervention and control villages of the MAMOP study. However, the design and conduct of the ITN trial makes it very unlikely that there was a differential effect on the MAMOP study groups. Moreover, the observed differences in malaria parameters between the two surveys may also be explained by differences in malaria transmission intensities due to annual variations in the pattern and amount of rainfall.

Another important observation from this study is the significant increase in clinically relevant CQ resistance in the study area over the two years observation period. This finding supports similar findings from a number of other studies which looked at the efficacy of CQ in Burkina Faso in recent years [18,19]. However, the fact that a more intense CQ drug pressure in the intervention villages was not associated with a more rapid increase of *in vivo *CQ resistance lends support to the hypothesis that better compliance with the full course of antimalarial treatment will likely delay resistance development [12,29].

Obviously, all-cause mortality and cause-specific mortality would have been the best outcome measure for this kind of intervention study. Due to sample size considerations and costs moderate to severe anaemia was chosen as the primary endpoint. The MAMOP community intervention had no effect on the prevalence of anaemia. This could at least partly be explained by the finding from another cohort study in the rural CRSN study area which has demonstrated that malnutrition but not malaria is the main determinant of anaemia development in young children of the area [30]. However, as the MAMOP study showed also no effects on other malaria parameters the likely explanation is that the intervention was simply not effective due to contamination, pre-existing differences in the coverage of malaria treatment in both groups and the unexpected rapid development of CQ resistance in the area.

Only a few studies have tried to measure the effects of complex malaria treatment interventions at the community level in endemic areas. One study in Ethiopia has measured the effectiveness of treating malaria episodes of young children through their mothers on mortality and was able to show a major reduction in all-cause mortality and malaria-specific mortality attributed to the intervention [15]. In a comparable study in Burkina Faso the intervention was associated with a significant reduction in malaria morbidity, but mortality was not measured [14]. However, other studies conducted in Kenya, The Gambia and Zaire and which were using village health volunteers for community malaria treatment were unable to show sufficient effects of the intervention on morbidity and mortality in young children [31-34].

In conclusion, this study has shown the feasibility of a complex malaria community intervention which has bridged the gap between the health workers at the peripheral health centres and the mothers of young children in individual households through women groups. The coverage of malaria treatment has substantially improved during the trial. Although such an approach is promising given the continuous lack of access to formal health services in much of rural SSA, there was no major difference in effectiveness between intervention and control areas. A possible intervention effect may have been masked by contamination, pre-existing differences in the coverage of malaria treatment in both groups, and the rapid rise in CQ resistance in the study area. Future studies should try to avoid confounding influences of other malaria interventions, employ more effective malaria first-line drugs such as artemisinin-based combination therapies (ACT), and preferably use mortality as the primary endpoint.

## Authors' contributions

BK, FS, AJ, OM, RS and GT designed the study. FS, BC and BK were responsible for the conduct of the study in Burkina Faso. FS, OM and HB analysed the data. All authors contributed to the interpretation of the data, helped write the paper, and read and approved the final manuscript.
